# Dosimetric comparison of sequential planning (SP) versus bias dose planning (BDP) in volumetric modulated arc therapy (VMAT)‐based breast radiotherapy boost

**DOI:** 10.1002/acm2.70544

**Published:** 2026-04-15

**Authors:** Thirumal Mani, Palanivelu Duraikannu, Chandramouli Ramalingam, Ajay Kumar Kondeti

**Affiliations:** ^1^ Department of Radiations Oncology All India Institute of Medical Sciences (AIIMS), Bibinagar Hyderabad Telangana India; ^2^ Department of Radiotherapy Manipal Hospitals Bengaluru Karnataka India

**Keywords:** bias dose plan, breast, sequential plan, and volumetric modulated arc therapy

## Abstract

**Aim/purpose:**

This study aims to evaluate and compare the dosimetric parameters of breast cancer radiotherapy plans using Volumetric Modulated Arc Therapy (VMAT), specifically contrasting the use of the bias dose planning (BDP) feature with sequential planning (SP) without bias in the Monaco Treatment Planning System (TPS).

**Materials and methods:**

A total of twenty left breast carcinoma patients were randomly selected for this study, which followed a two‐phase treatment regimen. In Phase 1, a dose of 40 Gy was delivered using the Elekta Versa HD, and the treatment plans were generated with 6 MV beams in the Monaco TPS using the VMAT technique. Phase 2 involved a sequential boost, delivering an additional 12.5 Gy, also using VMAT. For each patient, two treatment plans were created for Phase 2: one utilizing the BDP feature and the other SP, allowing for a direct dosimetric comparison of target volume, Organ at risk (OAR), normal tissue exposures, conformity index (CI) and Homogeneity index (HI).

**Results:**

Both the BDP and SP achieved comparable target volume coverage, with similar conformity and homogeneity indices. However, BDP demonstrated better sparing of OARs. Specifically, BDP significantly reduced the mean doses to the left lung (1496.4 ± 47.9 cGy vs. 1594 ± 189.3 cGy), right lung (465.9 ± 109.1 cGy vs. 591.6 ± 251.4 cGy), and heart (770.1 ± 232.0 cGy vs. 970.2 ± 65.5 cGy) compared to SP (all *p* < 0.05). Additionally, volume‐based metrics, including V20 and V5Gy, consistently favored BDP. There was no substantial difference in the absolute volumes (cc) of the Body‐PTV structure receiving 10% to 90% of the prescription dose between the BDP and SP plans, and the differences were not statistically significant (*P* ≥ 0.05). These findings underscore the dosimetric advantage of BDP in minimizing radiation exposure to critical structures without compromising target coverage.

**Conclusion:**

The integration of the bias dose feature in VMAT‐based breast cancer radiotherapy planning offers dosimetric advantages by improving sparing of critical organs without compromising target coverage or plan quality. This study highlights the importance of incorporating bias dose planning (BDP) in multiphase breast radiotherapy, especially when sequential boosts are required and supports its suitability for adoption in routine clinical practice.

## 1 INTRODUCTION

Breast cancer remains the most common malignant tumor among women and continues to be a leading cause of cancer‐related mortality.^1^, ^2^, ^3^ Currently, adjuvant radiotherapy (RT), particularly whole‐breast irradiation (WBI) following breast‐conserving surgery (BCS), is widely accepted as the standard of care for patients with early‐stage breast cancer. This multimodal treatment approach has been shown to enhance local control and improve overall survival rates.^4^, ^5^ Additionally, delivering a tumor bed boost after WBI has demonstrated a further reduction in local recurrence.^6^ In recent years, advanced radiation therapy techniques such as VMAT have been adopted to achieve optimal target dose coverage while minimizing radiation exposure to surrounding healthy tissues, offering an alternative to the conventional three‐dimensional conformal radiotherapy (3D‐CRT) approach. As VMAT has evolved, various techniques with increasingly sophisticated arc designs have emerged and are now competing for prominence in the treatment of breast cancer.^7^

An additional boost to the tumor bed in WBI treatment planning is considered a critical component for most patients following BCS,^8^ as it can significantly improve local control, particularly in those with poor prognostic factors.^9^, ^10^ Among the commonly employed boost techniques are Sequential Boost (SEQ) and Simultaneous Integrated Boost (SIB). Of these, SIB has demonstrated advantages such as shorter overall treatment time, improved dose distribution, and greater patient convenience, leading to better treatment tolerance.^11^, ^12^ However, there is currently a lack of comprehensive studies comparing the dosimetric outcomes of SEQ and SIB techniques. Some studies have indicated that SEQ plans offer better target coverage for both the breast and boost volumes, along with lower mean heart doses. However, other studies suggest that SIB provides improved conformality and superior dose distribution.^13^ Bias dose planning (BDP) is a feature available in some TPS that allows a previously calculated dose distribution (base plan) to be incorporated into the optimization of a subsequent plan. By accounting for the cumulative dose during optimization, this method reduces the risk of hot spots and excessive dose overlap in normal tissues, while maintaining adequate coverage of the planning target volume (PTV). Such an approach is particularly relevant in SEQ planning, where accurate summation of whole‐breast and boost doses is critical to achieve both tumor control and organ sparing. Therefore, this study aims to evaluate the dosimetric parameters of the PTVs, as well as critical organs and normal tissues receiving radiation during VMAT treatment planning for breast radiotherapy (RT).

## 2 MATERIALS AND METHODS

A total of twenty patients diagnosed with left breast carcinoma were randomly selected for this study. Following the outpatient department (OPD) consultation, each patient underwent the immobilization process. The patient was positioned on a breast board at an angle suitable for their comfort, and aligned using sagittal lasers on the mould room couch. A thoracic thermoplastic cast was used to immobilize the breast region, ensuring maximum separation of the contralateral (opposite) breast from the ipsilateral (treated) breast. The patient's arms were raised above the head and secured by holding arm poles. Subsequently, the patient was transferred to the CT simulator room for imaging required for RT planning. The patient setup in the simulator room replicated the mould room positioning. Surgical scar markings were identified using lead wires, and CT fiducial markers were placed with the aid of room lasers (Gammex, Sun Nuclear). CT imaging was performed using a Somatom Confidence 16‐slice CT simulator (Siemens Healthcare GmbH, Germany), covering the region from the lower mandible to the upper abdomen, with a slice thickness of 3 mm. Upon completion of the CT simulation, the acquired images were transferred to the Monaco Treatment Planning System (TPS) through the hospital's network for further RT planning.

### 2.1 Treatment planning (TPS)

The CT images were imported into the Monaco TPS, where target delineation and contouring were performed. The Gross Tumor Volume (GTV), Clinical Target Volume (CTV), PTV, and all OARs were contoured by the radiation oncologist in accordance with NCCN guidelines. In the first phase of treatment, a dose of 40 Gy was prescribed in 16 fractions. This was followed by a SEQ to the tumor bed in the second phase, with an additional prescribed dose of 12.5 Gy delivered in 5 fractions.

### 2.2 Phase‐I plan

**FIGURE 1:**
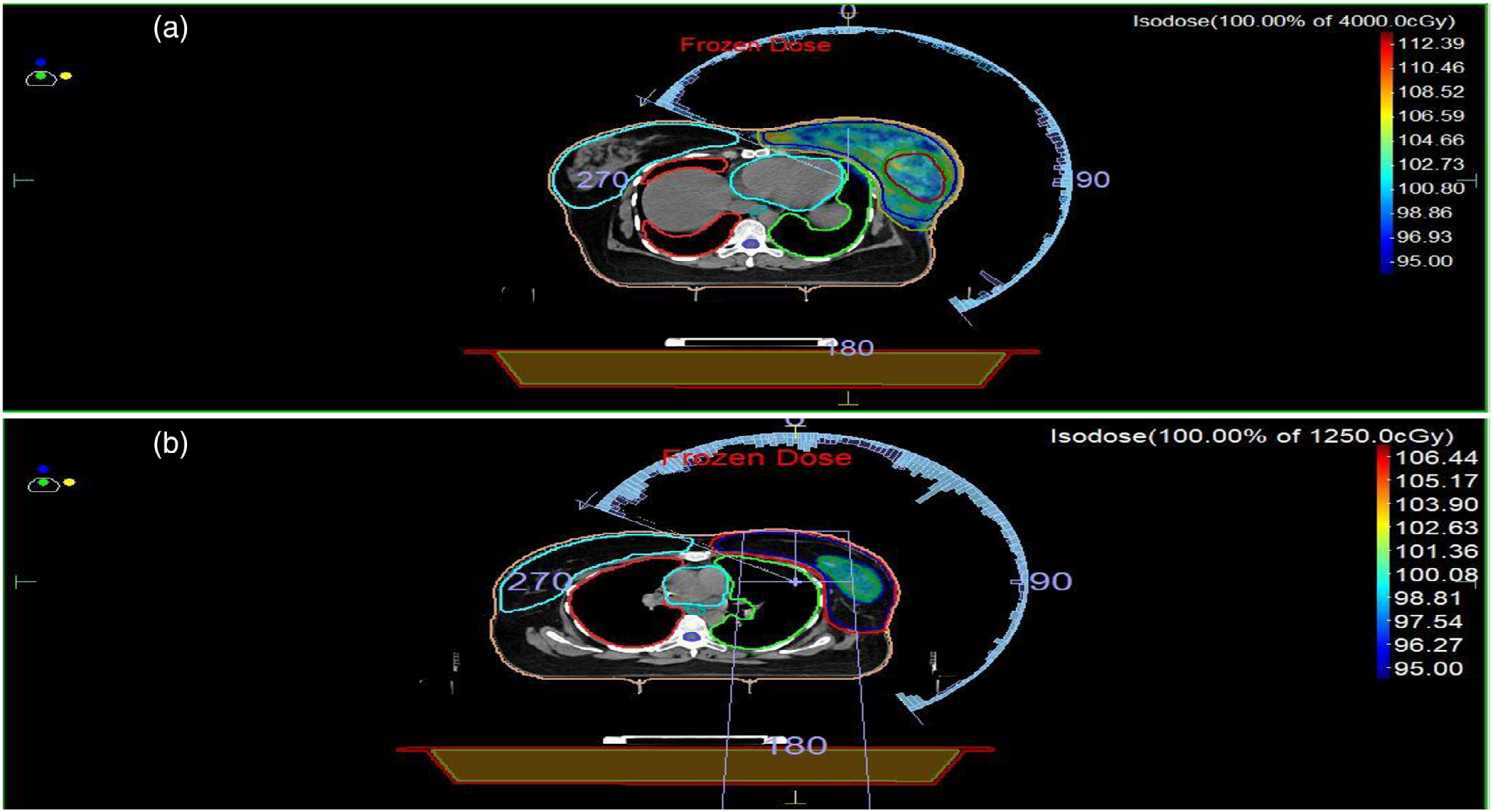
(a) VMAT Arc arrangements of Phase 1 plan 40 Gy. (b) VMAT Arc arrangements of Phase 2 boost plan 12.5 Gy.

VMAT planning was performed using the Monaco TPS, Version 13.5 for delivery on the Elekta Versa HD linear accelerator. Two coplanar arcs were utilized, with gantry angles ranging from 320° ± 15° to 140° ± 20°, as illustrated in Figure 1(a). An auto flash margin of 2 cm was applied in the IMRT parameters, and a 6 MV photon beam was used for dose calculation. In Phase 1 plan included both the breast and supraclavicular (SC) regions within a single treatment plan, without using a separate arc exclusively for the SC area.

Cost functions were defined based on the PTV and OARs to achieve the prescribed dose of 40 Gy in 16 fractions. The Monte Carlo algorithm was employed for VMAT optimization. The plan was iteratively optimized to improve dose distribution and ensure an optimal final treatment plan.

### 2.3 Phase ‐II without bias dose plan

Boost plans were generated in the same Planning System using the same parameters that is beam energy, segmentation width, target and surface margins, and collimator angle as used in Phase 1 plan, Only the arc angles were reduced specifically targeting the tumor bed. The arc angles ranged from 345° ± 5° to 110° ± 10°, as shown in Figure 1(b). IMRT and sequential planning parameters were maintained as in Phase I, with a prescribed dose of 12.5 Gy delivered in 5 fractions. Cost functions were assigned based on the PTV and OARs, and optimization was performed once. Upon completion of the boost plan, a plan sum was created by combining the Phase I and Phase II plans, and the cumulative dose distribution was evaluated for a total dose of 52.5 Gy.

### 2.4 Phase ‐II with bias dose plan

**FIGURE 2:**
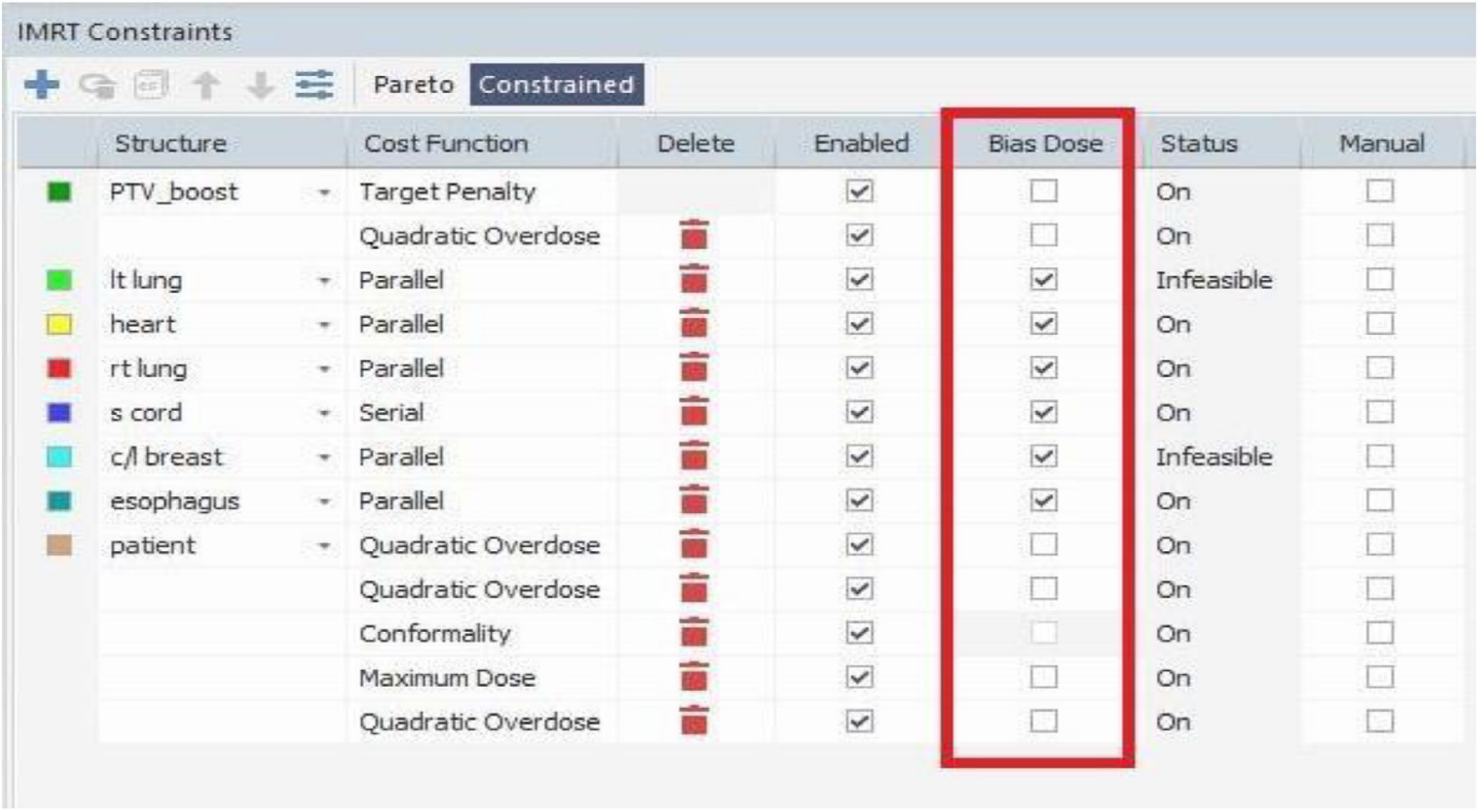
Treatment planning system (TPS) image showing the use of the bias dose option. During plan optimization. This feature allows incorporation of a previously calculated dose distribution into the optimization process, ensuring accurate cumulative dose evaluation. The optimization workspace displays the objective functions applied to the target and OARs, with bias dose settings highlighted.

A boost plan delivering 12.5 Gy in 5 fractions was created using the bias dose feature in the Monaco TPS, based on the Phase I plan. A VMAT technique was employed using a coplanar arc with reduced arc angles, similar to the Phase‐II plan without the bias dose plan. The bias‐dose optimization technique was applied only for the breast boost phase II, which was planned as a continuation of the initial breast +SC plan. Cost functions were assigned to the Boost PTV and relevant OARs. In the optimization window, the bias dose option was disabled (unchecked) for the PTV and Body, while it remained enabled for all OARs, as illustrated in Figure 2. In this context, the term “infeasible” indicates that certain optimization constraints were relaxed by the planning system to prevent PTV under dosage. Because the left lung and contralateral breast are located close to the target, higher optimization priority was assigned to these organs to meet their dose limits, and the reported infeasibility reflects this prioritization within the Monaco optimization process. In this BDP workflow, the dose distribution from the completed Phase 1 whole‐breast plan (40 Gy) was used as the base dose for the Phase 2 boost plan optimization. The base plan was imported into the Monaco TPS and incorporated during optimization to ensure that the cumulative dose from both phases was accurately considered. This allowed the optimizer to recognize regions already receiving higher doses in Phase 1 and appropriately adjust the boost dose distribution, thereby minimizing overlap and avoiding excessive dose to normal tissues. The entire planning process followed a sequential two‐step approach: (1) creation and finalization of the Phase 1 whole‐breast plan, and (2) generation of the Phase 2 boost plan with or without incorporation of the bias dose feature. The plan was then optimized accordingly. The maximum iteration limit and planning time were kept consistent across all plans to ensure comparability between approaches. For the bias dose plan, beams were re‐initialized before optimization, and the process was performed independently of the SP. This ensured that improvements observed with the bias dose approach reflected the optimization strategy itself, rather than extended iterations or continuation of a previous solution. Since the bias dose technique incorporates the initial dose distribution, evaluation of the cumulative dose of 52.5 Gy was performed directly on the bias dose plan, eliminating the need to create a separate plan sum.

In our clinical workflow, the radiation oncologist evaluates each plan individually, including Phase I (whole‐breast) and Phase II (boost), as well as the final plan sum (composite dose distribution) before treatment approval.

Both treatment plans aimed to achieve optimal target coverage, with the goal of ensuring that CTV Breast V95% and PTV Breast V95% received at least 95% of the prescribed dose. Similarly, CTV Boost V95% and PTV Boost V95% were also required to receive a minimum of 95% of the prescribed dose. Additionally, the volume of PTV receiving more than 107% of the prescribed dose (PTV V107%) was restricted to less than 5%, and the maximum dose to the PTV (PTV Dmax) was constrained to not exceed 110% of the prescription dose.

The conformity and homogeneity of the dose distribution were evaluated using the following indices:

Conformity Index (CI) = PIV/TV, where *PIV* is the Prescription Isodose Volume and *TV* is the Tumor Volume.

Homogeneity Index (HI) = (D2%–D98%)/DP where *D2%* is the dose received by 2% of the target volume, *D98%* is the dose received by 98% of the target volume and *DP* is the prescribed dose.

Various dosimetric parameters were evaluated for the ipsilateral lung, contralateral lung, heart, contralateral breast, and spinal cord. For the ipsilateral lung, the percentage volumes receiving 5 Gy (V5), 20 Gy (V20), and the mean dose were analyzed. For the heart, the volume receiving 25 Gy (V25) and the mean dose were assessed. In the case of the contralateral lung, both V5 and the mean dose were evaluated. For the contralateral breast, only the mean dose was considered.

Additionally, a structure termed Body–PTV was generated by subtracting the PTV from the external body contour. This volume was used to assess the extent of low‐dose radiation spread (ranging from 10% to 90% of the prescribed dose) in healthy tissue a significant concern in VMAT techniques.

## 3 RESULTS

A detailed dosimetric comparison of the PTV and OARs for the Bias Dose Plan (BDP) and the non‐Bias Dose or SP is presented in Table 1. This table summarizes quantitative metrics related to target coverage and OAR sparing, providing a comprehensive overview of both planning approaches.

**TABLE 1:** Dosimetric comparison between bias dose plan and non‐bias dose plan.

Structure	Parameter	Bias dose plan (Mean ± SD)	Non‐bias dose plan (Mean ± SD)	*P* value
PTV boost	V95% Max dose (cGy) Mean dose (cGy) Conformity index (CI) Homogeneity index (HI)	97.6 ± 2.0 5515.6 ± 73.6 5240.1 ± 87.4 0.97 ± 0.02 1.04 ± 0.02	99.4 ± 1.1 5638.3 ± 60.5 5285.2 ± 91.5 0.99 ± 0.009 1.07 ± 0.01	0.011 0.006 0.003 0.015 0.037
PTV breast	V95% Max dose (cGy) Mean dose (cGy) Conformity index (CI) Homogeneity index (HI)	97.5 ± 0.9 5515.6 ± 73.6 4253.14 ± 83.05 0.97 ± 0.01 1.36 ± 0.03	97.9 ± 0.76 5638.3 ± 60.5 4304.6 ± 126.2 0.97 ± 0.006 1.357 ± 0.04	0.009 0.006 0.012 0.65 0.63
Left lung	V20 (%) V5 (%) Mean dose (cGy)	28.19 ± 5.1 91.3 ± 6.4 1496.4 ± 47.9	29.02 ± 5.19 92.7 ± 5.96 1594 ± 189.3	0.021 0.227 0.016
Right lung	V5 (%) Mean dose (cGy)	38.1 ± 13.1 465.9 ± 109.1	41.37 ± 13.72 591.6 ± 251.4	0.008 0.017
Heart	V25 (%) Mean dose (cGy)	1.0 ± 0.3 770.1 ± 232.0	1.3 ± 0.5 970.2 ± 65.5	0.016 0.010
Opposite breast	Mean dose (cGy)	436.4 ± 121.7	447.1 ± 120.4	0.007
Spinal cord	Max dose (cGy)	2100.3 ± 485.4	2112.2 ± 485.9	0.045
Body‐PTV (dose spillage)	Percentage of prescription dose 10% 20% 30% 40% 50% 60% 70% 80% 90%	Volume receiving in (cc) 35.4 ± 4.8 18.1 ± 2.7 11.6 ± 2.0 8.0 ± 1.5 5.4 ± 0.97 3.5 ± 0.67 1.7 ± 0.5 0.199 ± 0.2 0.025 ± 0.04	Volume receiving in (cc) 35.8 ± 4.5 18.3 ± 2.6 11.7 ± 1.9 8.1 ± 1.4 5.5 ± 0.9 3.6 ± 0.7 1.9 ± 0.6 0.3 ± 0.2 0.1 ± 0.01	≥0.05

**FIGURE 3:**
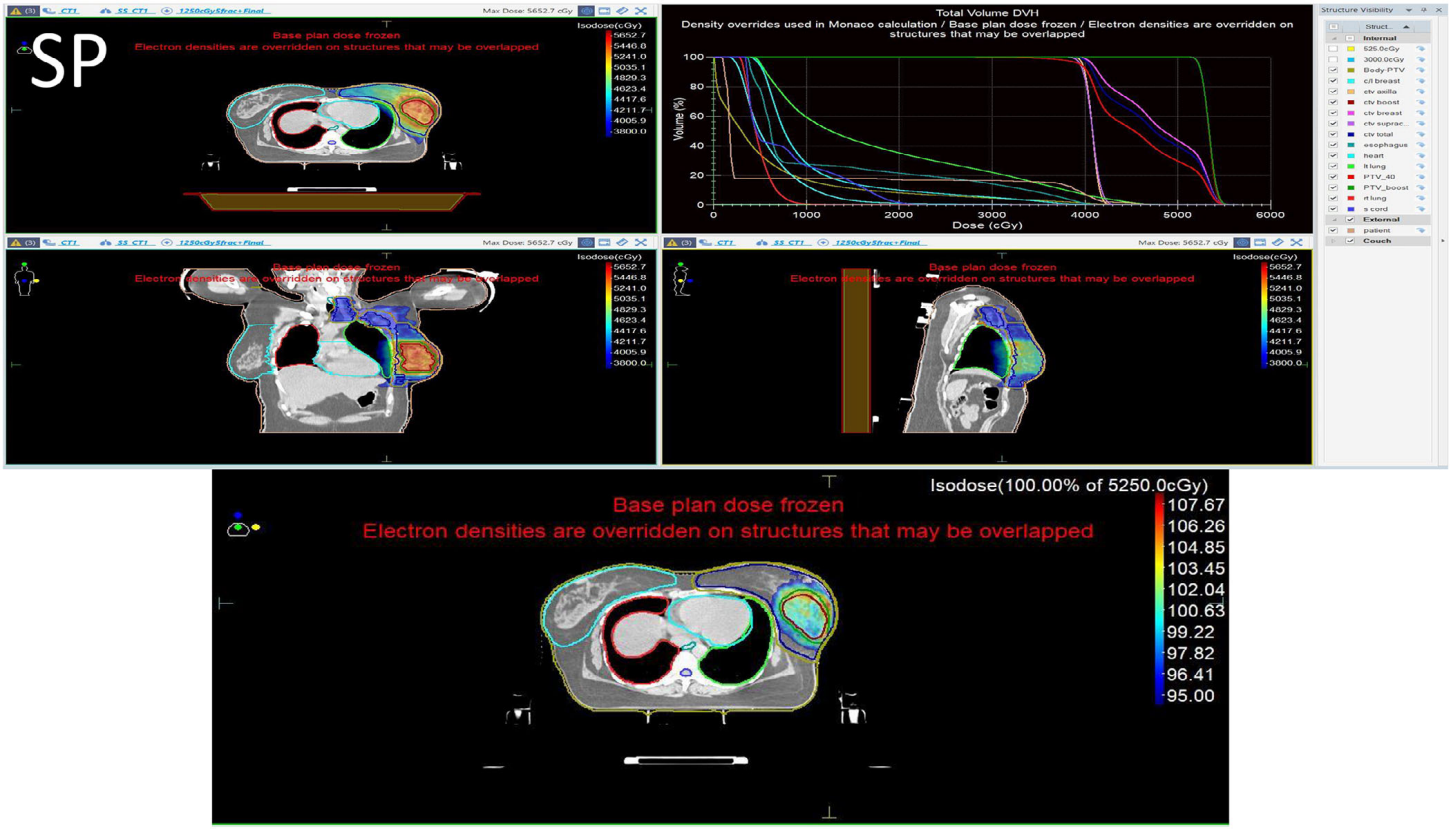
Dose distribution and DVH of the SP. The color wash represents minimum 95% of 40 Gy/for the PTV breast and minimum 95% of 52.5 Gy for the PTV boost, demonstrating both whole‐breast and boost coverage. Axial, sagittal, and coronal views are shown with PTV and OAR contours. The dose scale legend indicates the corresponding isodose levels.

**FIGURE 4:**
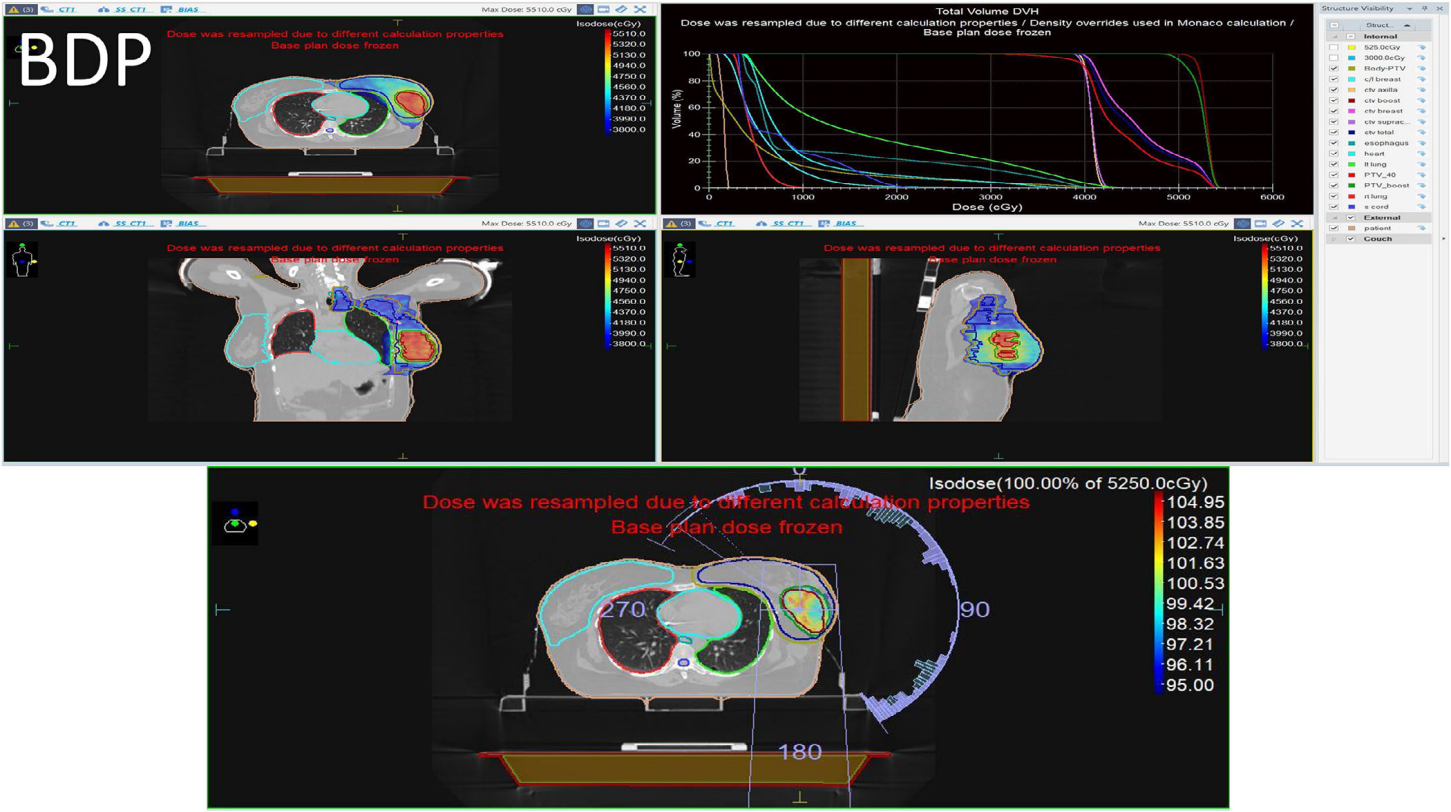
Dose distribution and DVH of the BDP. The color wash represents minimum 95% of 40 Gy for the PTV breast and minimum 95% of 52.5 Gy for the PTV boost, demonstrating both whole‐breast and boost coverage. Axial, sagittal, and coronal views are shown with PTV and OAR contours. Thedose scale legend indicates the corresponding isodose levels.

**FIGURE 5:**
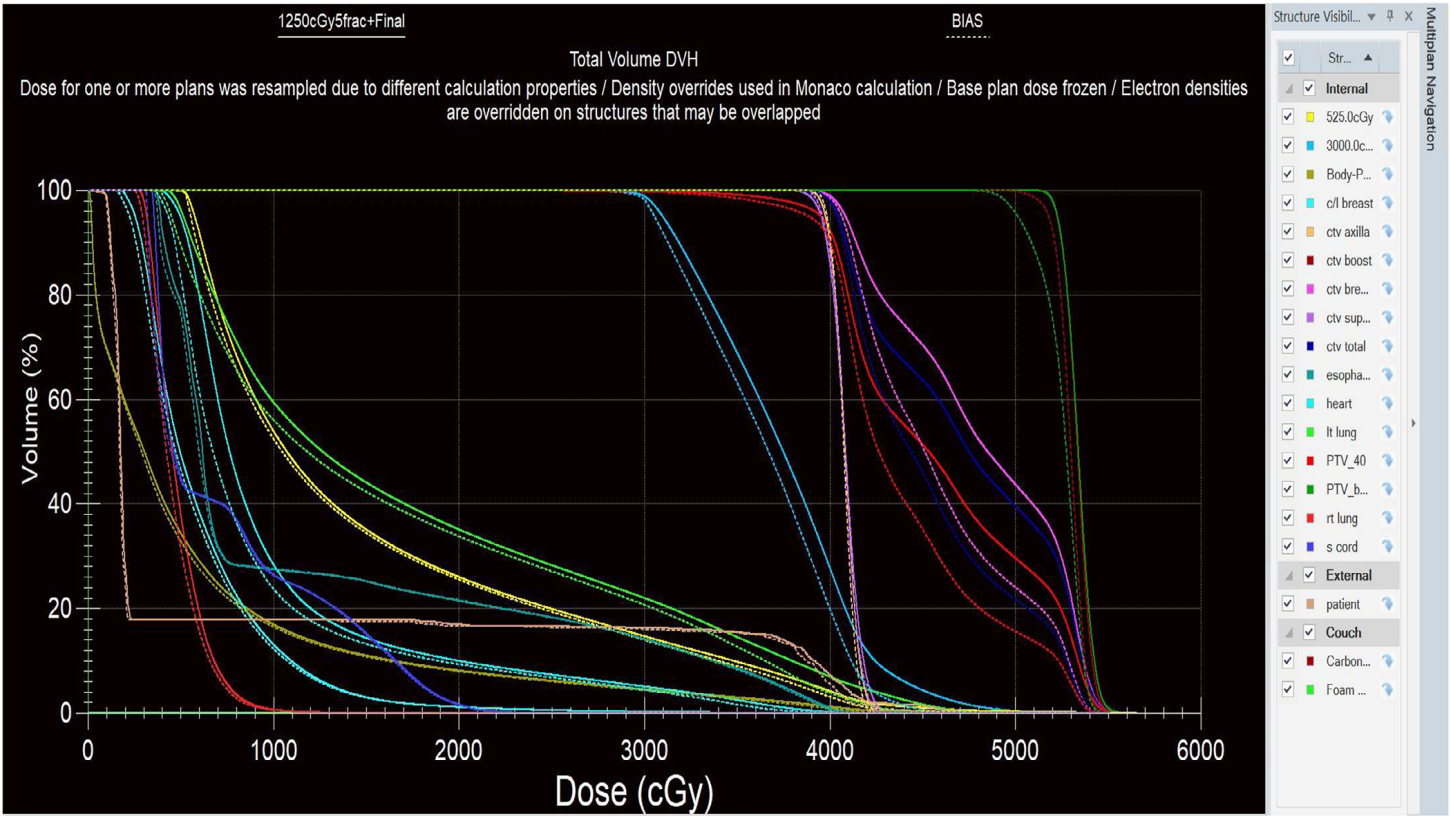
DVH comparison between the “BDP” plan (dotted lines) and “SP”plan (solid lines). The plots show dose–volume relationships for the PTV, GTV, and OARs.

Figures 3 and 4 visually demonstrate the dose distribution within the patient anatomy in coronal, sagittal, and transverse planes. These figures show the 95% isodose coverage of the target volumes using isocolor wash displays, allowing clear visualization of spatial dose conformity. The accompanying Dose Volume Histograms (DVHs) further illustrate the dose distribution to both the target and OARs, offering insight into plan quality from a volumetric perspective. Figure 5 provides a graphical comparison between the BDP and SP techniques, highlighting differences in dosimetric parameters across the structures of interest.

In terms of target volume coverage, both BDP and SP techniques achieved comparable results. The CI and HI values were similar for both plans, although the differences in CI and HI reached statistical significance in Table 1, the absolute numerical variations were small and both plans remained within clinically acceptable limits. A slight but consistent reduction in PTV D95 was observed in the BDP plans compared to SP. however, all predefined clinical target coverage criteria were satisfied, indicating that the observed variation did not compromise clinical acceptability. PTV Breast dose indices also showed comparable coverage and clinically acceptable CI and HI values for both techniques indicating effective and uniform dose distribution within the target volume. Additionally, low‐dose spillage to surrounding healthy tissues was minimal and consistent between the two plans.

However, a distinct advantage of the BDP approach was observed in the sparing of critical organs. The BDP consistently resulted in reduced radiation exposure to several OARs, with statistically and clinically significant improvements in both mean dose and volume‐based parameters. In the left lung, the mean dose (Dmean) was reduced from 1594 ± 189.3 cGy in the SP plan to 1496.4 ± 47.9 cGy in the BDP plan (*P* < 0.05). The volume receiving 20 Gy (V20) was also lower in the BDP plan (28.19 ± 5.1%) compared to the SP plan (29.02 ± 5.19%) (*P* < 0.05). No statistically significant difference was observed in the low‐dose volume metric V5Gy for the left lung between BDP and SP plans (*P* > 0.05).

For the right lung, a statistically significant difference was observed in both mean dose and low‐dose volume. The BDP plan achieved a mean dose of 465.9 ± 109.1 cGy, compared to 591.6 ± 251.4 cGy with the SP plan (*P* < 0.05). Similarly, the V5Gy was lower in the BDP plan (38.1 ± 13.1%) than in the SP plan (41.37 ± 13.72%) (*P* < 0.05).

In the heart, which is especially critical in left‐sided breast irradiation, the BDP plan significantly lowered the mean dose to 770.1 ± 232.0 cGy, compared to 970.2 ± 65.5 cGy with the SP plan (*P* < 0.05). This reduction is of clinical relevance due to the potential risk of radiation‐induced cardiac toxicity.

Additionally, in the contralateral breast, a minor yet consistent reduction in the mean dose was observed with the BDP plan (436.4 ± 121.7 cGy) when compared to the SP plan (447.1 ± 120.4 cGy), with the difference reaching statistical significance (*P* < 0.05), suggesting a modest improvement in limiting unnecessary radiation exposure to the unaffected breast tissue.

And the analysis for Body‐PTV structure volumes receiving various dose levels revealed no substantial difference between the BDP and SP plans. Specifically, the absolute volumes (measured in cc) receiving dose levels ranging from 10% to 90% of the prescription dose were found to be similar across both planning techniques. Statistical evaluation confirmed that these differences were not significant (*P* ≥ 0.05).

## 4 DISCUSSION

This study assessed the dosimetric performance of BDP in comparison to conventional SP within the framework of VMAT for breast RT, using the Monaco TPS. The analysis focused on the boost of a two‐phase treatment approach for breast cancer, in which WBI was followed by a SEQ boost to the tumor bed. The results indicate that BDP provides advantages in sparing OARs, while effectively maintaining target coverage, dose conformity, and homogeneity. The application of BDP facilitated the incorporation of Phase‐I dose distributions into the optimization process of the Phase‐II plan. This integration enabled a more comprehensive and informed planning approach by considering the cumulative dose delivered during the initial WBI. In contrast, conventional SP generates the boost plan independently of the primary phase, which may lead to suboptimal dose distributions and unnecessary radiation exposure to adjacent normal tissues. The results of this study demonstrated that BDP consistently reduced both mean dose and volume‐based parameters (V5Gy, V20Gy) in critical structures, including the heart, lungs, and contralateral breast, thereby enhancing OAR sparing without compromising target coverage. Although the mean PTV dose was marginally lower in BDP than SP, both achieved ≥95% V95% coverage of the prescribed dose, confirming that target coverage remained clinically equivalent between the two techniques. The shift in the DVH shoulder around 4000 cGy reflects expected differences in cumulative whole‐breast dose distribution between SP and BDP, as the BDP incorporates the Phase‐I base dose during optimization. Importantly, PTV Breast V95% ≥ 95% was achieved in both techniques, confirming equivalent target coverage.

The mean heart dose was significantly lower with the BDP at 770.1 ± 232.0 cGy compared to the SP, which recorded 970.2 ± 65.5 cGy. This reduction is clinically significant for patients with left‐sided breast cancer, where long‐term cardiac toxicity remains a well‐documented concern.^14^, ^15^. Additionally, BDP resulted in a lower mean dose (Dmean) and V20Gy to the left lung, reflecting improved lung sparing and a potentially reduced risk of radiation pneumonitis. Although the right lung and contralateral breast also experienced dose reductions with BDP, the benefits were comparatively modest, likely due to the enhanced dose modulation provided by the BDP approach.

In our study, both plans met the clinical acceptance criteria, ensuring that 95% of the PTV received at least 95% of the prescribed dose (V95% ≥ 95%) and that the maximum dose did not exceed 110% of the prescription. Hence, the delivered dose to the target is biologically equivalent between the two approaches. The slightly lower mean PTV dose in the BDP plans reflects improved dose uniformity rather than underdosage, consistent with ICRU recommendations.

The advantage of BDP lies in its ability to guide the optimizer using the prior dose distribution from the whole‐breast (Phase 1) base dose plan during the boost (Phase 2) optimization, thereby improving conformity of the cumulative plan. By integrating the base dose into optimization, regions of potential overdose to normal tissues can be minimized while ensuring adequate PTV coverage across all phases. In contrast, non‐bias optimization treats each phase independently, which may lead to overlap of high‐dose regions or reduced conformity in the composite plan. Our findings support the rationale that bias dose incorporation improves overall plan quality by balancing tumor coverage. Our findings align with previous studies that underscore the importance of dose optimization in multiphase RT. Earlier research on hybrid or composite planning strategies has demonstrated enhanced plan quality when prior dose distributions are incorporated into subsequent phases of treatment.^16^, ^17^ However, limited work has specifically investigated the application of the BDP tool within the Monaco TPS. This positions our study among the first to systematically evaluate its effectiveness compared to conventional planning approaches in breast cancer cases. The observed reduction in dose spillage to normal tissues further supports the potential of BDP to minimize the cumulative dose burden on healthy structures throughout extended treatment courses.

An additional benefit observed was the reduced inter‐patient variability in organ dose metrics with BDP, suggesting that the technique provides more consistent and predictable dosimetric outcomes. From a clinical workflow perspective, the integration of BDP did not significantly increase planning complexity or time, and its implementation was straightforward within the existing Monaco environment. Although planner time was not formally recorded in this study, the BDP process is not more time‐consuming than SP, as it builds on the existing base plan and reduces preliminary setup steps This further supports its feasibility for routine clinical adoption.

Despite the promising results, this study has several limitations. The sample size was limited to twenty patients, which may restrict the generalizability of the findings. Additionally, only dosimetric parameters were assessed; clinical outcomes such as toxicity, local control, and long‐term cardiac or pulmonary effects were not evaluated. Future studies with larger cohorts and prospective clinical follow‐up are warranted to validate the observed dosimetric benefits and correlate them with patient outcomes.

In summary, the use of BDP in VMAT‐based breast RT offers better dosimetric advantages over conventional SP. By incorporating prior dose distributions into the boost phase, BDP enhances OAR sparing without compromising target coverage or plan quality. These findings support the routine use of BDP in multiphase breast RT protocols and highlight the importance of dose‐aware planning in achieving optimal therapeutic ratios.

## 5 CONCLUSION

The integration of the bias dose feature in VMAT‐based breast cancer radiotherapy planning offers dosimetric advantages by improving sparing of critical organs without compromising target coverage or plan quality. This study highlights the importance of incorporating BDP in multiphase breast RT, especially when SEQ boosts are required and supports its suitability for adoption in routine clinical practice.

## AUTHOR CONTRIBUTIONS

All authors contributed significantly to this work. Thirumal M and Ajay Kumar Kondeti conceptualised the study, supervised the project, and led manuscript writing. Thirumal M and Palanivelu D performed data collection and analysis. Chandramouli R contributed to methodology design and manuscript editing. Ajay Kumar Kondeti and Chandramouli R provided clinical oversight and critical revisions. All authors reviewed and approved the final manuscript.

## CONFLICT OF INTEREST STATEMENT

The authors declare no conflicts of interest.

## Data Availability

The data and materials of this original research article will be made available to the public upon request.
